# A global empirical typology of anthropogenic drivers of environmental change in deltas

**DOI:** 10.1007/s11625-016-0357-5

**Published:** 2016-03-19

**Authors:** Zachary D. Tessler, Charles J. Vörösmarty, Michael Grossberg, Irina Gladkova, Hannah Aizenman

**Affiliations:** 1 Environmental Sciences Initiative, CUNY Advanced Science Research Center, City University of New York, New York, USA; 2 Department of Computer Science, City College of New York, New York, USA

**Keywords:** Deltas, Risk, Vulnerability, Environmental change

## Abstract

It is broadly recognized that river delta systems around the world are under threat from a range of anthropogenic activities. These activities occur at the local delta scale, at the regional river and watershed scale, and at the global scale. Tools are needed to support generalization of results from case studies in specific deltas. Here, we present a methodology for quantitatively constructing an empirical typology of anthropogenic change in global deltas. Utilizing a database of environmental change indicators, each associated with increased relative sea-level rise and coastal wetland loss, a clustering analysis of 48 global deltas provides a quantitative assessment of systems experiencing similar or dissimilar sources and degrees of anthropogenic stress. By identifying quantitatively similar systems, we hope to improve the transferability of scientific results across systems, and increase the effectiveness of delta management best practices. Both *K*-Means and Affinity Propagation clustering algorithms find similar clusters, with relative stability across small changes in *K*-Means cluster number. High-latitude deltas appear similar, in terms of anthropogenic environmental stress, to several low-population, low-latitude systems, including the Amazon delta, despite substantially different climatic regimes. Highly urbanized deltas in Southeast Asia form a distinct cluster. By providing a quantitative boundary between groups of delta systems, this approach may also be useful for assessing future delta change and sustainability given projected population growth, urbanization, and economic development trends.

## Introduction

With highly dynamic geomorphology, coastal river deltas are particularly sensitive to perturbations of natural delta processes by anthropogenic activities. Human actions in both the upstream watershed and the coastal zone, as well as offshore, can have short- and long-term effects on delta sustainability (Ericson et al. 2006; Syvitski et al. 2009; Renaud et al. 2013). Deltas rely on a regular supply of sediment to counterbalance land subsidence and relative sea-level rise due to natural delta processes of sediment compaction (Syvitski and Saito 2007). A wide range of human activities influence the mobilization, delivery, deposition, and erosion of sediment to and from a delta. In this study, we use estimates of these anthropogenic factors from global datasets to classify 48 delta systems within a typology of delta environmental stress.

For the nearly half-billion people living on deltas, the rate of relative sea-level rise (RSLR), the combination of land subsidence and sea-level rise, is a crucial factor for long-term sustainability. Given the low relief typical of coastal delta systems, small levels of RSLR can result in substantial coastline migrations (Sarwar and Woodroffe 2013; Stanley and Clemente 2014). Over the past several decades, a number of deltas in wealthy nations have embraced large-scale engineering efforts in an attempt to protect their communities from coastal and fluvial flooding (Day et al. 2007; VanKoningsveld et al. 2008). However, this approach is expensive and likely unsustainable on its own (Day et al. 2014; Tessler et al. 2015). Alternative solutions must be developed to address the drivers of RSLR. The identification of best practices and exchange of knowledge across delta boundaries is critical for the protection of livelihoods and maintenance of river deltas as population centers. Here, we identify commonalities among deltas with respect to environmental drivers of RSLR, and develop new tools for descriptive statistical analysis of common environmental stresses on deltas. By explicitly outlining the similarities among deltas, we hope to reduce the challenge inherent in managing many individual deltas, each with respect to their unique characteristics, and strengthen avenues for collaboration and sharing of effective management strategies.

Major challenges in conducting comparative research across deltas include differences in data availability and differences in scale. Prior to the remote sensing era, Earth science was limited to in situ datasets. While still the gold standard in terms of ground-truth and flexibility, these datasets are inherently limited in time and space. In situ data collection methods do not scale well spatially, for comparative delta assessment, or temporally, for trend identification. Remote sensing and numerical modeling provide consistent information about the Earth’s surface over larger geographic regions and longer time spans than is practical from in situ approaches alone. Here, we use these complementary tools to develop a globally consistent database of delta environmental stresses that contribute to RSLR and loss of protective coastal wetlands. Over time, RSLR contributes to a given delta’s fluvial and coastal flood risk, in conjunction with a delta’s exposure to hazardous events and the social vulnerability of the delta population (Brooks et al. 2005; Tessler et al. 2015). Using a suite of indicators that have been identified as having direct or indirect effects on delta RSLR (Ericson et al. 2006; Syvitski et al. 2009; Vermaat and Eleveld 2013), we define a global typology of delta environmental stress and present metrics for assessment of the typology. The typology is based on exploratory data analysis methods and highlights patterns in environmental stress affecting global deltas. Improved understanding of the common sources of anthropogenic impacts on deltas may better enable researchers and practitioners across delta systems to share management strategies in support of long-term sustainability.

## Materials and methods

**Fig. 1:**
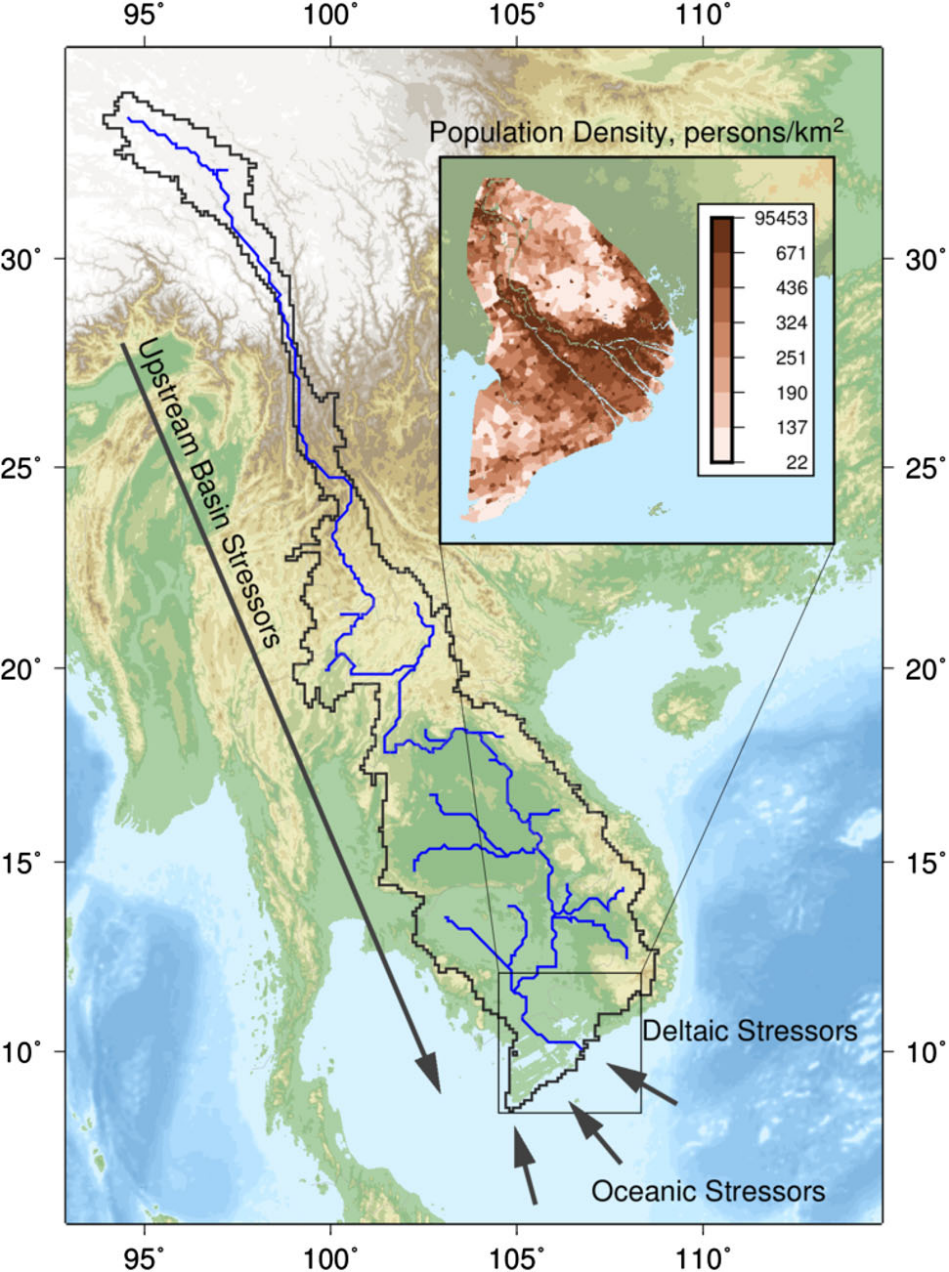
The Mekong River Delta, representative of the domains considered in this study. The delta is impacted from anthropogenic stressors acting in the upstream basin and transmitted to the delta via the river network, as well as local stresses such as population density (*inset*), and oceanic stressors, such as sea-level rise

Anthropogenic change variables are selected to be indicative of processes that affect the rate of delta RSLR, or the health of coastal wetlands (Table 1). These processes occur in three domains: the upstream river network, the coastal delta, and the offshore marine environment (Fig. 1). Aggregated indicator estimates were extracted over 48 major river deltas (Table 2) from global remote sensing products and numerical modeling outputs. Delta extent maps were defined using topography, river networks, fluvial soil maps, and visual inspection of vegetation patterns in Landsat optical remote sensing imagery (Ericson et al. 2006). Upstream watershed extents were defined based on the Simulated Topological Network (STN06) digital river network (Fekete et al. 2001).

**Table 1:** Delta environmental indicators used in this study

Indicator	Collection method	Scale	Reference
Population density (delta, basin)	Administrative data	2.5 arc-min	GPWv3 (CIESIN 2005)
Reservoir volume sediment trapping	Administrative data, numerical model	Vector database, 6 arc-min river network model	GWSP-GRanD (Lehner et al. 2011), WBMplus (Wisser et al. 2010)
Wetland disconnectivity (delta, basin)	Remote sensing, map digitization	0.5°	Vörösmarty et al. (2010)
Impervious surface area (delta, basin)	Remote sensing	1 km	Elvidge et al. (2007)
Groundwater depletion	Administrative data, numerical model	0.5°	Wada et al. (2012)
Oil and gas extraction	Literature review, geologic assessment, administrative data	GIS vector maps	USGS (2000) World Petroleum Assessment

Several indicators as noted are aggregated separately in the coastal delta and the upstream river basin. Other indicators are only aggregated over the delta. Collection method indicates the original methodology used to collect the data, prior to any synthesis of higher level data products. Scale refers to the spatial resolution of the final data product

**Table 2:** Cluster labels from the five clustering algorithms used in this study

Delta	Affinity propagation	*K*-Means 6	*K*-Means 7	*K*-Means 8	*K*-Means 9
Amazon	1	1	1	**1**	1
Amur	1	4	4	**1**	1
Brahmani	8	6	6	**6**	9
Burdekin	1	1	1	**1**	1
Chao Phraya	7	6	6	**7**	6
Colorado	4	4	4	**4**	4
Congo	3	3	3	**3**	3
Danube	6	2	2	**8**	8
Dnieper	7	2	2	**2**	8
Ebro	4	4	4	**4**	4
Fly	3	3	3	**3**	3
Ganges	6	6	6	**6**	6
Godavari	7	6	6	**7**	6
Grijalva	2	2	2	**2**	2
Han	5	5	5	**5**	5
Hong	5	5	5	**5**	5
Indus	2	2	2	**2**	2
Irrawaddy	6	2	6	**6**	6
Krishna	7	6	6	**7**	6
Lena	1	1	1	**1**	1
Limpopo	8	4	2	**8**	8
Mackenzie	1	1	1	**1**	1
Magdalena	8	5	5	**8**	5
Mahakam	3	3	3	**3**	3
Mahanadi	8	6	7	**8**	9
Mekong	7	6	6	**6**	6
Mississippi	2	2	2	**2**	2
Moulouya	1	4	4	**4**	4
Niger	2	2	2	**2**	2
Nile	7	6	7	**7**	7
Orinoco	3	3	3	**3**	3
Parana	4	4	4	**4**	4
Pearl	5	5	5	**5**	5
Po	6	2	6	**6**	6
Rhine	5	5	5	**5**	5
Rhone	5	5	5	**8**	5
Rio Grande	4	4	4	**4**	4
Sao Francisco	2	4	2	**2**	2
Sebou	8	6	7	**7**	7
Senegal	2	4	4	**4**	4
Shatt-el-Arab	2	4	4	**4**	4
Tana	2	4	2	**2**	2
Tone	7	6	6	**6**	6
Vistula	8	6	7	**8**	9
Volta	2	2	2	**2**	2
Yangtze	5	5	5	**5**	5
Yellow	2	2	2	**2**	8
Yukon	1	1	1	**1**	1

Deltas that share a cluster number within a column are classified in the same cluster by that algorithm. Specific numerical labels are arbitrary, though matching cluster labels have been assigned to similar clusters of deltas in each algorithm

The *K*-Means 8 cluster assignments, in bold, are used to define the typology in this study

In the upstream river network, the most important processes are those that affect the fluxes of sediment and freshwater transported downstream through the river network. We include four indicators from the upstream basin: reservoir sediment retention, impervious surface area, wetland disconnectivity, and population density. The presence and size of artificial dams and reservoirs are major factors in the anthropogenic conditioning of river networks (Vörösmarty et al. 2003), and are likely to increase in importance with new dams under construction or planned in the Mekong basin (Kuenzer et al. 2012) and around the globe (Zarfl et al. 2014). Water storage in large, still reservoirs behind dams provides time and space for sediment to settle to the riverbed, reducing sediment concentrations downstream of the dam (Vörösmarty et al. 2003). Using the Global Water System Project—Global Reservoir and Dam Database (Lehner et al. 2011), we estimate the average residence time of water in artificial reservoirs within the basin as the total volume of reservoirs within each watershed, normalized by the mean river discharge at the basin mouth, as simulated by the global hydrological model WBMplus (Wisser et al. 2010). This model computes a daily water balance in each 6 arc-min grid cell over the global land surface. Surface runoff is routed through a digital river network transport model to estimate mean discharge entering the delta apex. Higher residence times in artificial reservoirs are associated with increased sediment retention (Vörösmarty et al. 2003) and reduced fluvial sediment fluxes to deltas (Syvitski et al. 2005).

The construction of impervious surfaces in the upstream watershed impacts run-off dynamics, with impervious surface area associated with reduced sediment mobilization (Pappas et al. 2011). In the delta itself, new sediment aggradation occurs in areas where sediment-laden river water is allowed to flood, depositing sediment and increasing the land elevation. Urban and agricultural land is often protected from flooding by constructed levees and dikes, which prevent freshwater and sediment inputs from reaching wetlands. Impervious surfaces constructed for some human purpose tend to be prevented from flooding, increasing the area’s long-term susceptibility to land subsidence. Impervious surface area is aggregated over basin and delta extents from global remote sensing estimates (Elvidge et al. 2007). This dataset is a global, 1-km resolution product based primarily on Defense Meteorological Satellite Program nighttime light observations, and calibrated against 30-m resolution estimates of impervious surface area for the conterminous USA developed by the USGS from Landsat observations.

Wetland disconnectivity is a measure of wetland area in use for agricultural or urban purposes. In many deltas, wetland loss is associated with construction of dikes and levees for flood control. Estimates of wetland disconnectivity are based on land area designated as wetlands in the Global Lakes and Wetlands Database (Lehner and Döll 2004), and occupied by agricultural or urban land use (Vörösmarty et al. 2010). Agricultural land cover is estimated from MODIS and GLC2000 datasets, and impervious surface area, described above, is used as a proxy for urban development. These wetlands are presumed to have lost much of their natural functioning, with extensive river channelization and levee construction to protect urban areas from flooding. Wetland areas disrupted by human use in many cases no longer provide the physical, biological, or chemical ecosystem services of natural systems. In some cases the wetlands are drained and the conversion is complete. In deltas, functioning natural wetlands provide a suite of services, including retention of fluvial sediment, and also act as a coastal buffer, reducing flood risk due to storm surges (Temmerman et al. 2012; Das and Crépin 2013) and coastal sediment erosion (Day et al. 2007).

Population density is taken as an indirect indicator of overall human pressure on watershed and coastal systems. We use Global Population of the World (GPWv3) data from 2000, and associated projections of 2015 population. Results with the higher resolution Global Rural–Urban Mapping Project (GRUMPv1) are similar, though only available through 2000. In the delta domains, we compute a modified population density that includes the population within a 25-km buffer around the delta. This incorporates environmental pressure from nearby population centers that depend on the delta for ecosystem goods and services. For instance, this broadened population density estimate includes the populations of Ho Chi Minh City and Karachi in the anthropogenic impact on the Mekong River Delta and Indus River Delta, respectively. In most other deltas, the marginal land is somewhat less populated than the delta itself, and the buffering reduces the mean population density. The reduction of measured population density for the majority of deltas has little effect on the final scores other than to improve the quality of the Mekong and Indus population density estimates. The 25 km size of the buffer is an order-of-magnitude estimate of the spatial size of Ho Chi Minh City and Karachi. Comparison of several buffers, from 5 to 100 km, suggests that small differences in buffer size have only minor impacts on resulting population density rankings. In addition to perturbation of sediment distribution processes, high population density also can have a direct impact on land subsidence: observations in the Fraser River delta have linked increased relative sea-level rise to the additional load from urban construction over delta sediments (Mazzotti et al. 2009).

We also consider the effect of fossil fuel and groundwater extraction from delta sediments. These processes reduce pore pressure in previously deposited sediments, resulting in accelerated sediment compaction (Morton et al. 2005). Land subsidence related to groundwater extraction has been associated with aquaculture activities in the Yellow Delta using *InSAR* observations, though it is likely that other deltas in Southeast Asia with growing aquaculture industries also are, or will be, affected (Higgins et al. 2013). Groundwater-related land subsidence has been directly associated with increases in local rates of flooding in the Po Delta (Carminati and Martinelli 2002). In this case, the groundwater has been used primarily for large-scale agricultural and industrial usage. We estimate groundwater extraction in excess of recharge from a global dataset (Wada et al. 2012) which was constructed using a combination of country-level groundwater abstraction statistics and hydrologic model-estimated recharge rates. In areas where recharge rates are high, it is assumed that the groundwater resources can sustainably support larger abstraction rates. Likewise, low recharge rates suggest that less groundwater can be sustainably removed before having an impact on land subsidence. Fossil fuel extraction can have a similar effect as groundwater pumping: in the Mississippi River delta, hydrocarbon extraction is an important driver of subsidence-related wetland loss, with historical subsidence rates of 8–12 mm/year, compared with 1–5 mm/year over the past 5000 years (Morton et al. 2005). For this study, we identify hydrocarbon extraction in deltas using data from the USGS World Energy Assessment (2000).

Local sea-level rise is a direct component of RSLR, and is estimated from satellite altimetry and tide gauge trends (Church et al. 2004). Several physical processes contribute to rising local sea levels; we include all processes that result in a long-term trend at the coastline. Both eustatic sea-level rise, a global component resulting from increases in ocean water volume due to ice sheet melting or reductions in the total volume of the ocean basins, and local rise due to long-term changes in ocean heat content or currents are included in the assessment. We do not remove trends due to glacial isostatic adjustment or the inverse barometer effect from the altimetry record as our interest lies in the total change in sea level relative to land, rather than solely the ocean water volume component of that rise. Isostatic adjustment, while not anthropogenic in origin, is an important component of RSLR, and directly affects wetland loss and coastline migration.

Several of these indicators, in particular basin reservoir trapping, span orders of magnitude across deltas. Excluding the Mahakam and Fly deltas, with no upstream dams in the GRanD database, basin reservoir trapping, estimated by mean residence time in reservoirs, ranges from 0.08 × 10^9^ s in the Congo River watershed, to 1005 × 10^9^ s in the Colorado River watershed. Due to the wide range of magnitudes, we rank and unit-normalize the data across all the deltas in the study. While rank-normalization eliminates the relative scale of extreme features, such as the very high reservoir residence time in the Colorado watershed, it also prevents the extreme values from effectively under-weighting a given indicator for all other deltas. In this way, we maintain potentially important information over the full data distribution.

**Fig. 2:**
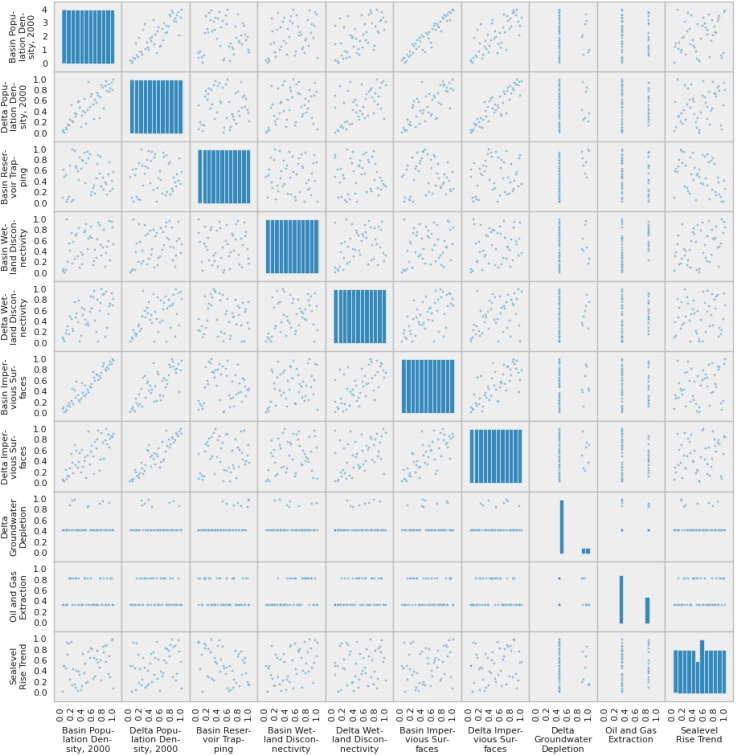
Scatter matrix of normalized environmental indicators extracted from the delta, upstream, and offshore domains.* Diagonal plots* show histograms for individual indicators, with the* vertical axis* representing frequency. Off the diagonal are scatter plots between pairs of indicators. Data are rank-normalized across all deltas. Several indicators (delta groundwater depletion, oil and gas extraction) do not span the 0–1 range due to tied ranks, handled by assigning the average rank to all tied deltas. Groundwater depletion has many zero-valued deltas where groundwater extraction does not appear to be in excess of recharge rates. Oil and gas extraction is a binary flag indicator, where the values zero and one indicate the absence and presence, respectively, of hydrocarbon extraction within the delta. Several environmental indicators show correlations, which are quantified using Spearman rank correlation coefficients in the main text. These indicators include both population density and both impervious surface area indicators

With important exceptions, most normalized indicators are only weakly correlated with each other (Fig. 2). Those exceptions are the basin and delta population density and impervious surface estimates. Impervious surfaces and population density are strongly correlated, with a Spearman rank correlation coefficient of 0.90 within deltas, and 0.95 within upstream basins. Population density in the upstream basin and the delta have a correlation coefficient of 0.84, while upstream and basin impervious surface estimates have a correlation coefficient of 0.74. While these four features are well correlated, the mean correlation coefficient across all other features is 0.14.

**Fig. 3:**
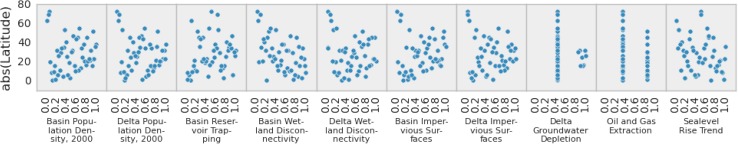
Relationships between each environmental indicator and the absolute value of delta latitude, taken as a proxy for climate. Each* dot* represents one delta. Weak relationships are found between latitude and both population density and impervious surface area indicators, where mid-latitude deltas have the highest population density and impervious surface area. Low- and high-latitude deltas tend to be less populated and developed, though the overall variability explained by latitude is low. Additionally, all deltas with unsustainable groundwater depletion are found within a narrow latitude band in the subtropics

Climate is not directly included in the set of environmental indicators. While climate has a major influence on the natural functioning of these systems, climate itself is not an anthropogenic driver of change. Sea-level rise, associated with anthropogenic change within the climate system, is included as an indicator in the study. Additionally, climate is an important component of how anthropogenic disturbances influence natural delta functioning and coastal risk (Tessler et al. 2015). Latitude, here taken as a proxy for climate, is not strongly associated with the environmental indicators (Fig. 3). Delta and basin population densities, and those indicators that are correlated with population such as impervious surface area are weakly related to latitude. At low and very high latitudes population density is low, while somewhat higher at mid-latitudes. However, there is substantial variability in the indicators that cannot be directly ascribed to latitude.

**Fig. 4:**
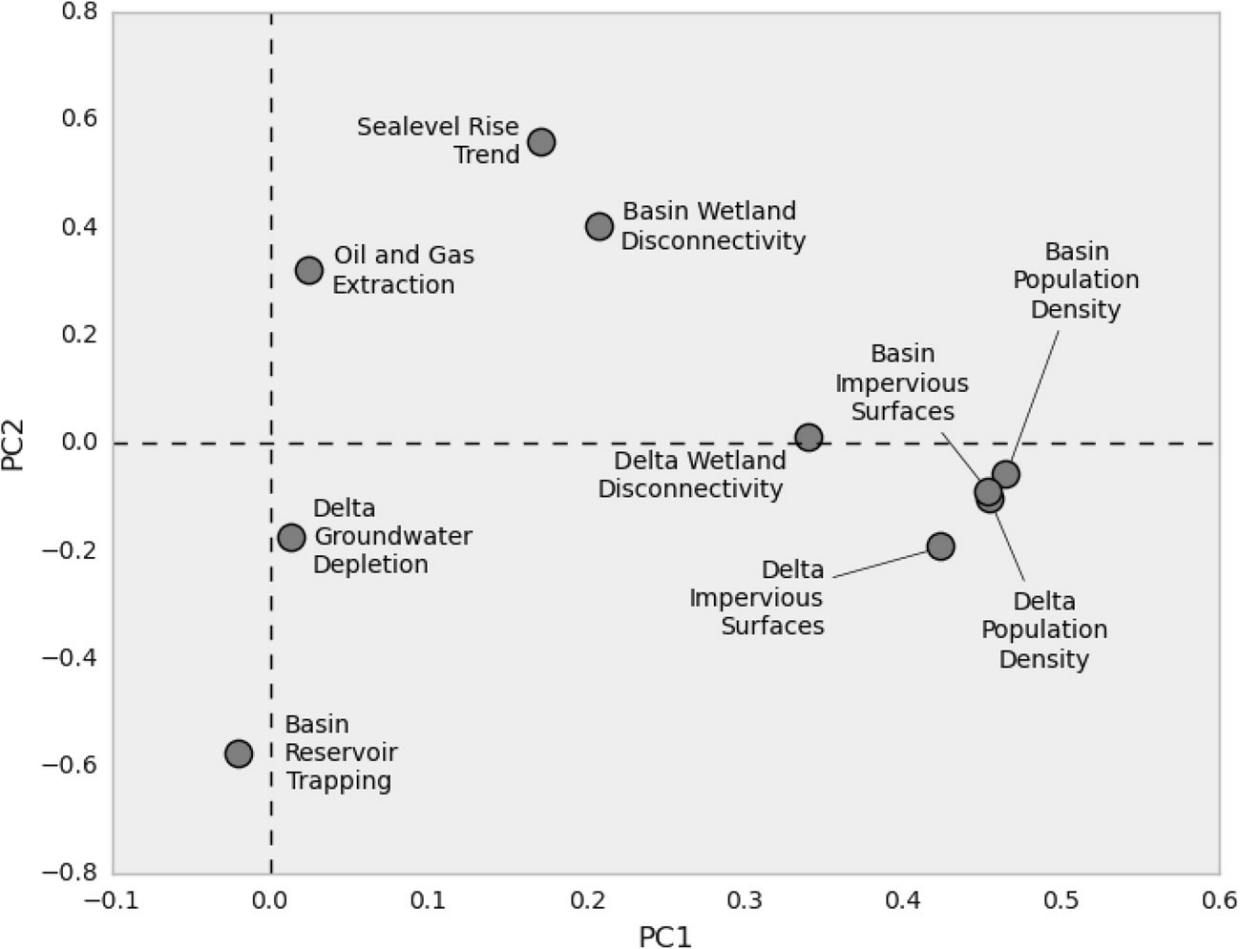
Principal component analysis loadings. Each indicator axis is projected into the first two components. The population density and impervious surface indicators are well correlated, and their projections are close in the two-dimensional PCA space

We choose to work with the observed variables, rather than extracting latent variables though Principal Component Analysis (PCA), to improve the interpretation of results. Using PCA, however, we can nonetheless examine the linear relationships between indicators (Fig. 4). Reduced to two dimensions, the correlated population and impervious surface indicators are all loaded on the first principal component. Delta wetland disconnectivity is also loaded on the first principal component, though not closely grouped with the population and impervious surface data. Hydrocarbon extraction, groundwater depletion, and reservoir trapping are loaded predominately on the second principal component, while sea-level rise and wetland disconnectivity in the upstream basin have positive loadings for both of the first two components.

Also important to note are the highly modal data for the groundwater and hydrocarbon indicators. The majority of the deltas in the study do not rely heavily on groundwater extraction in excess of recharge rates, and their groundwater indicator scores are zero. In rank-normalized space, their scores tie at zero, and rather than the ranks ranging from 1 to 40, each delta is assigned the middle rank of 20.5. The remaining eight non-zero, non-tied deltas are assigned ranks 41–48. Similarly, since the hydrocarbon extraction dataset provides a binary flag indicating only the presence or absence of oil or gas activities within a delta, there are 31 deltas tied at zero, and 17 deltas tied at one. Deltas with hydrocarbon extraction activity, therefore, are all ranked 16, and those without hydrocarbon activity are ranked 40. Resulting unit-normalized scores are either 0.33 or 0.83.

## Results

Cluster analysis is used to identify common patterns of environmental stress among deltas and define a delta environmental typology. Several clustering methods have been described in the literature; here we contrast results using the *K*-Means (KM) and Affinity Propagation (AP) methods (MacQueen 1967; Frey and Dueck 2007). Each delta, characterized by the 10 basin, coastal, and offshore indicators, is described by a vector in **R** ^10^, and assigned to the nearest of *K* cluster means according to the minimum Euclidean distance. Each cluster mean is then changed to match the centroid of its clustered data points. This process of data point reassignment to the nearest mean, and cluster mean readjustment to the centroid is repeated until the algorithm converges. While straightforward, the algorithm suffers from several drawbacks. Convergence to a local minimum is guaranteed; however, this may not be the global optimum. The final clustering is sensitive to the initial points chosen for the *K*-means. Finally, the number of clusters, *k*, must be explicitly specified. Initialization and convergence issues can be addressed using cluster ensembles. To provide guidance in the choice of *k*, we also identify clusters using the AP method. This is a message-passing algorithm, where data points are chosen as “exemplars,” or cluster centers, based on their similarity to other data points. Nearby points, in **R** ^10^, are considered similar. In an iterative process, candidate “exemplars” are chosen to maximize similarity among all clusters. A key strength of the AP algorithm is that the number of clusters does not need to be specified, but rather are an output of the clustering process.

AP clustering identifies eight distinct clusters. We compare these clusters with a set of KM clusterings with values of *k* ranging from 6 to 9. Each KM clustering is computed as the best clustering, in terms of the minimum mean distance between samples and cluster centers, from 1000 random initializations of cluster centers. While the numeric labels used to identify a given cluster within an AP or KM clustering are arbitrary, we have numbered the labels to highlight similar delta clusters across each AP or KM clustering. The delta membership for each clustering scheme is presented in Table 2.

Identification of the optimal clustering is a non-trivial task, and a substantial literature (e.g., Rousseeuw 1987; Halkidi et al. 2001; Meilă 2007) has been devoted to the development of metrics for assessing the quality or consistency of a given clustering. We compute silhouette scores, a measure of cluster density, as an estimate of the quality of a given clustering. For each sample, the silhouette score is defined as the ratio:

1 $$(b - a)/{ \hbox{max} }(a, b)$$

where *a* is the mean distance from the sample to all other intra-cluster samples, and *b* is the mean distance from the sample to all other samples in the next closest cluster. This ratio approaches 1 for “perfect” clusters where all samples are identical. Values near zero suggest that the sample belongs only marginally better in the assigned cluster than the next closest cluster, while negative scores indicate the sample is closer to a neighboring cluster than its own cluster. This metric assumes that small distance between samples is the defining characteristic of a cluster, the same assumption that the *K*-Means and Affinity Propagation algorithms use to define clusters. Average silhouette scores across all samples in a given clustering are shown in Table 3. Mean scores are similar across each algorithm at approximately 0.20, with slightly higher scores for KM8 and KM9. These positive, but low, scores suggest that the boundaries between some clusters are weakly defined.

**Table 3:** Mean silhouette scores for each clustering method

	AP	KM6	KM7	KM8	KM9
Silhouette score	0.17	0.19	0.20	0.21	0.21

Silhouette scores are a measure of how close a delta is to other deltas within its cluster, relative to deltas in the next nearest cluster. Scores range from −1, representing a poor clustering, to 1, representing a clustering where all deltas within each cluster perfectly align. Scores near zero are indicative of small separation between clusters

**Fig. 5:**
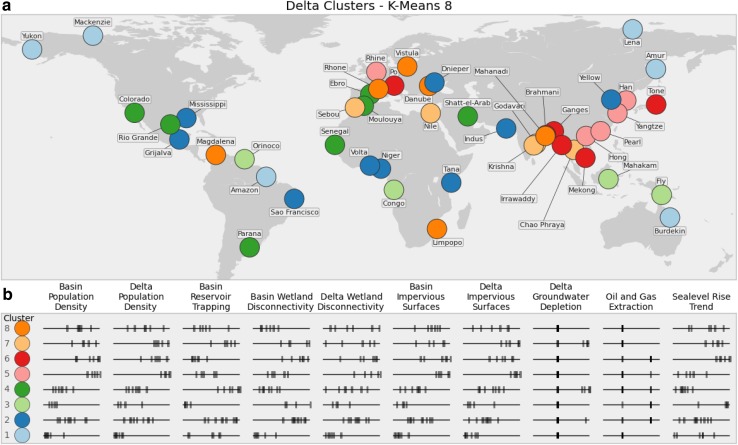
**a** Map of KM8 delta cluster membership. **b** Normalized indicator scores of the deltas in each cluster. *Vertical dashes* are positioned to show the normalized indicator values of the deltas within the given cluster, relative to the full range of deltas in the study. *Darker vertical dashes* indicate overlapping values

**Fig. 6:**
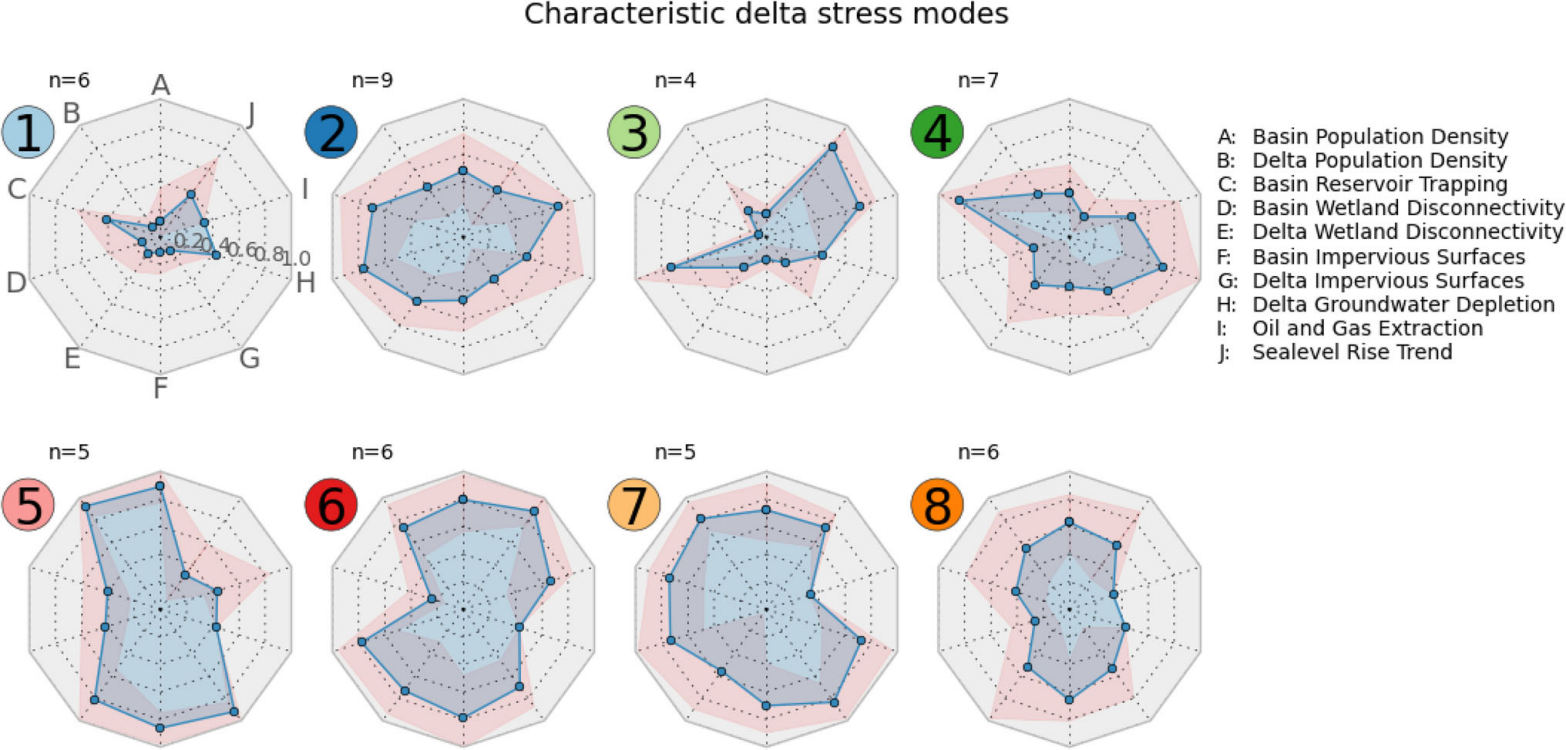
Characteristic delta environmental stress modes, clustered by KM8. Each diagram indicates the mean (*blue*) and the range (*pink*) of each indicator for deltas in that cluster.* Cluster 1* and* 3* contain deltas with relatively low overall environmental stress, while* Clusters 2, 4, and 8* have a wider range of stress levels across different indicators.* Clusters 5, 6,* and* 7* are predominately high environmental stress systems, though deltas in each are less stressed by certain indicators. Deltas in* Cluster 6*, for instance, have very little oil and gas extraction, and relatively low wetland disconnectivity in the upstream river basin

We focus here on results from KM8 and use this clustering to define the global deltas environmental typology. KM8 has the same number of clusters as the Affinity Propagation algorithm, with slightly better silhouette scores than the other clusterings. For several clusters, however, there is little to no difference in delta membership across algorithms. Sensitivity of each cluster to the clustering algorithm chosen is examined below. Geographic distribution of the clusters, or environmental types, are mapped in Fig. 5a, with the distribution of indicator scores among each type in Fig. 5b. Characteristic “fingerprints” for each type are shown in Fig. 6.

*Type 1* deltas (light blue) are characterized by very low anthropogenic stress in the local delta domain, and to a lesser degree in the upstream watershed as well. The deltas in this type are the Amazon, Amur, Burdekin, Lena, Mackenzie, and Yukon deltas. The four remote, high-latitude delta systems in the study are all in this type. All have very low delta and basin population densities. Indeed, the only moderate environmental indicator values among Type 1 deltas are elevated basin reservoir trapping values in the Amur, Burdekin, Lena, and Mackenzie deltas, and moderate sea-level rise trends in the Amazon, Burdekin, Lena, and Mackenzie deltas.

*Type 2* deltas (dark blue) are moderately stressed across most of the environmental indicators. These systems have mid-range population densities, both in the delta and the upstream basin. This type comprises the Dnieper, Grijalva, Indus, Mississippi, Niger, Sao Francisco, Tana, Volta, and Yellow deltas. These deltas are geographically dispersed, have moderate to high wetland conversion in the upstream watershed and coastal delta areas, and moderate to high volume of artificial reservoirs on the upstream river network. We note that additional environmental challenges can be highly important in specific deltas, such as soil salinization in the Indus Delta (Aslam and Prathapar 2006), and this methodology utilizes an approximate estimate of the current delta environmental states to develop clusters.

*Type 3* deltas (light green) have low populations in both spatial domains, but high wetland disconnectivity in the upstream basin, suggesting heavy conversion of wetland to agricultural use. Deltas in this type include the Congo, Fly, Mahakam, and Orinoco deltas. Located in the tropics, these deltas have low upstream reservoir trapping, and do not rely on groundwater extraction. Both of these characteristics reflect high freshwater input, as the reservoir volume indicator is normalized by river discharge. A consistent supply of freshwater obviates the need for unsustainable groundwater extraction to support agricultural or municipal use. The Fly, Mahakam, and Orinoco are exposed to very high local sea-level rise rates. Hydrocarbon extraction activities occur in the Congo, Mahakam, and Orinoco deltas as well.

*Type 4* deltas (dark green) are low to moderate population deltas, several located in the subtropics, and tend to be reliant on water engineering efforts. The deltas in this type are the Colorado, Ebro, Moulouya, Parana, Rio Grande, Senegal, and Shatt-el-Arab. Located primarily in arid regions, the upstream river networks feeding these deltas are heavily dammed, with a high mean water residence time in artificial reservoirs. Modeling results suggest that the Colorado, Rio Grande, Senegal, and Shatt-el-Arab all rely on unsustainable groundwater extraction from the delta. Local sea-level rise trends tend to be low for these deltas.

*Type 5* deltas (pink) are very densely populated and urbanized systems. This delta type includes the Han, Hong, Pearl, Rhine, and Yangtze. These are some of the most densely populated deltas in the world, and also have densely populated upstream basins. The populations are highly urban, with high impervious surface area. With the exception of the Rhine, oil and gas extraction is not common in these deltas. Reservoir trapping in the upstream basin is low to moderate, and groundwater extraction is not a major factor for deltas in this type.

*Type 6* deltas (red) are moderately to very highly populated, both in the delta and upstream watershed. Deltas comprising this type are the Brahmani, Ganges–Brahmaputra, Irrawaddy, Mekong, Po, and Tone. This type is similar to Type 5, though the upstream watershed has greater wetland disconnectivity, suggesting more agricultural conversion of wetlands. Additionally, this type is differentiated from Type 5 by substantially greater exposure to local sea-level rise. Hydrocarbon extraction occurs in four deltas in this type: the Brahmani, Ganges, Irrawaddy, and Po.

*Type 7* deltas (light orange) are highly populated and similar to Type 5, but with a greater reliance on upstream dams and local groundwater extraction for water management. This type includes the Chao Phraya, Godavari, Krishna, Nile, and Sebou deltas. Unlike Types 5 and 6, none of the deltas in this type have substantial hydrocarbon extraction activities, per the 2000 USGS World Energy Assessment. These deltas tend to be urbanized, with high impervious surface areas, similar to Type 5 and to a lesser extent Type 6.

*Type 8* deltas (dark orange) tend to be moderately populated, both in the upstream basin and the coastal delta. This type is similar to Type 2, with somewhat greater population density and greater local sea-level rise trends. Deltas in this type are the Danube, Limpopo, Magdalena, Mahanadi, Rhone, and Vistula. Relative to Type 2, deltas in Type 8 are also characterized by a lower likelihood of oil and gas extraction activity. Furthermore, deltas in this type have relatively low wetland disconnectivity scores in the upstream basin, suggesting more intact wetland systems in the contributing watershed.

## Discussion and conclusions

**Fig. 7:**
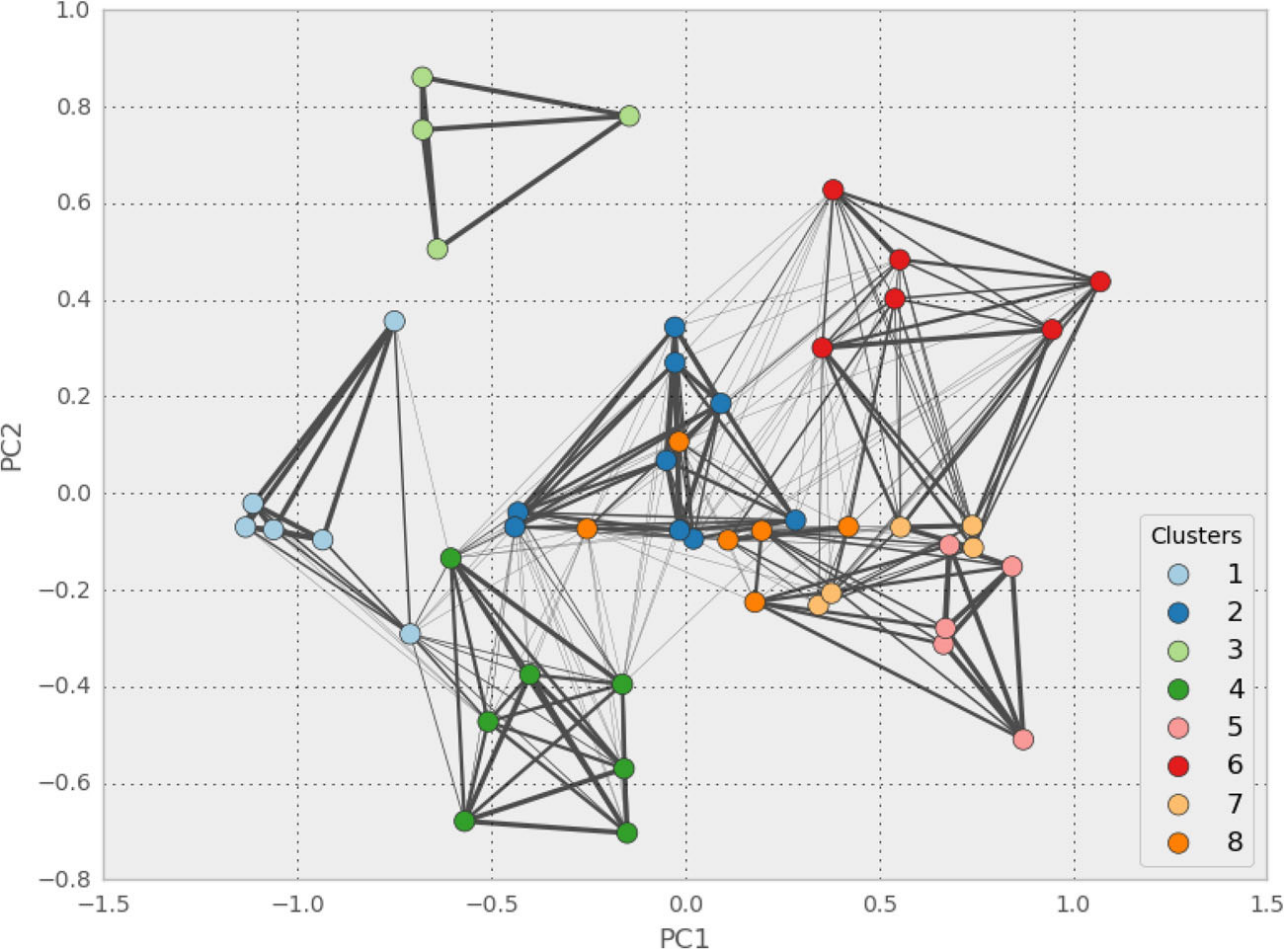
Deltas projected onto the first two principal components. *Colors* indicate KM8 cluster.* Line width* of edges between delta vertices scales with the number of algorithms which assign both deltas to the same cluster. The absences of a connecting edge between two delta vertices indicate that none of the clustering algorithms assign both of those deltas to a single cluster

These results provide a means for classifying global deltas by their exposure to particular environmental stresses. Based on the qualitative inspection of the types above, it appears that several of the types (Types 1, 3, 6) are well separated along several indicator dimensions, while others (Type 2, 8) have substantial overlap and are differentiated by only a few indicators. Examining the clusters projected into the first two PCA dimensions (Fig. 7), supports this finding. Type 3 appears well separated from other clusters, even in the reduced two-dimensional feature space. These are tropical, low population and development deltas with high sea-level rise exposure, and appear distinctly different from other deltas in the study. Type 1 deltas, predominately high latitude, also appear substantially different, though the Amazon and Amur, on the margin of this cluster, are somewhat similar to Types 3 and 4, respectively. Type 6 deltas are also well separated, in contrast to the overlap observed between Types 2 and 8, and Types 5 and 7. The differentiating characteristics of these clusters are found in higher dimensions than the two visualized in Fig. 7. By definition, these higher dimensions describe less variance in the dataset, and these overlapping clusters are less distinctive than those with greater separation in the first two PCA components. However, the observed overlap between clusters 2 and 8, 7 and 8, and 5 and 7 observed in Fig. 7 is increased by the visualization of the data in only two dimensions.

By examining the likelihood of any two deltas being clustered together, we can estimate the sensitivity of a cluster’s members to the algorithm chosen. In Fig. 7, the edges between the delta vertices indicate the number of clustering algorithms that place both deltas in the same cluster. Unconnected deltas are never clustered together, while the heaviest lines indicate the two connected deltas are always found in the same cluster. Again, Type 3, consisting of the Congo, Fly, Mahakam, and Orinoco, is highlighted as particularly distinct; these deltas are always clustered with each of the others, and never with any other deltas. Type 1, consisting of the Amazon, Amur, Burdekin, Lena, Mackenzie, and Yukon, appears relatively well separated from other clusters, though several of the clustering algorithms group the Amur delta with the Moulouya, Parana, Colorado, and others of Type 4.

This analysis also shows that the deltas of Type 6 and Type 7, despite their separation when projected into the first two PCA components, are frequently clustered together by the other clustering algorithms (AP, KM6, KM7, and KM9). The boundaries between Types 2 and 8, 7 and 8, and 5 and 7 are sensitive to the clustering method.

The diffuse boundaries between several of the delta types are less surprising given that these are fully independent systems whose attributes can continuously vary. That several delta types can be quantitatively distinguished suggests that some aspects of the environmental stresses on these systems are responding to common anthropogenic or natural dynamics. For instance, the stresses on high-latitude systems (Type 1) appear distinct from those in the tropics (Type 3) or arid subtropics (Type 4), despite no geographic input to the clustering. This analysis also highlights how delta population density, while an extremely important, first-order driver of environmental stress, should be considered in conjunction with other sources of environmental change. In particular, the challenges facing deltas in Type 6 are likely to be more difficult that those facing Type 5, despite similarities in high population density and urban extent, due to the additional influence of local sea-level rise trends.

There are several limitations to this delta environmental stress clustering methodology. The primary limitation is the requirement for global datasets, which eliminates the possibility of including data that have only been collected in specific deltas or regions. In this study, we rely on remote sensing and numerical models for the bulk of the indicators. We also utilize aggregate data over the entire delta, eliminating potentially rich heterogeneity across the delta. While we have examined the sensitivity of the clustering to the algorithms used, questions about data completeness remain, and how stable these clusters may prove to be with additional indicators.

Additional indicators of environmental stress that we have not included here would be required for a complete accounting of environmental impacts on deltas. These include important factors such as changes to wave erosion processes due to ice melt in high-latitude deltas, isolation of the delta plain from the river network by dikes, and soil salinization. However, while adding more data may bring this tool closer to a proxy for overall environmental impact, it will not necessarily improve the clustering. With more indicators, the dimensionality of the clustering becomes more of a challenge. As the 48 delta data points become more spread out in the high-dimensional indicator space, the clustering is likely to become less robust and meaningful.

Additionally, the current clustering methodology is not optimal for monitoring of changes to delta environmental sustainability. The clusters are insensitive to small changes in the environmental indicators due to the rank-normalization data processing. A delta indicator must change significantly enough to result in a change in rank before the normalized value reflects the increase or decrease in the raw indicator value. However, alternative normalizations may be used to increase sensitivity to change. Logarithmic or other non-linear normalizations may be useful, given the multiple orders of magnitude spanned by several of the indicators. The methodology is flexible enough to include additional indicators in future assessments.

It is clear that sustainable delta management requires a systems approach. Climate change induced sea-level rise increases coastal risk (Tessler et al. 2015), prompting an increased search for low-carbon energy, particularly hydropower (Zarfl et al. 2014). However, hydropower benefits come with significant and well-understood downstream consequences (Vörösmarty et al. 2003; Sun et al. 2012). The optimal balance between these and other trade-offs will vary across delta systems. Policy-makers need improved tools to weigh these options in the context of their particular delta system. Hopefully, the ability to look to “neighboring” deltas to test and share best practices, in an environmental stress space rather than solely a geographic space, will improve the decision-making process, and have positive consequences for the livelihoods of the growing populations living on the world’s coastal delta systems.
